# Therapeutic Potential of Resveratrol in COVID-19-Associated Hemostatic Disorders

**DOI:** 10.3390/molecules26040856

**Published:** 2021-02-06

**Authors:** Roberta Giordo, Angelo Zinellu, Ali Hussein Eid, Gianfranco Pintus

**Affiliations:** 1 Department of Medical Laboratory Sciences, College of Health Sciences, and Sharjah Institute for Medical Research, University of Sharjah, University City Rd, Sharjah 27272, United Arab Emirates; robertagiordo2000@yahoo.it; 2 Department of Biomedical Sciences, University of Sassari, 07100 Sassari, Italy; azinellu@uniss.it; 3 Department of Basic Medical Sciences, College of Medicine, QU Health, Qatar University, P.O. Box 2713 Doha, Qatar; 4 Biomedical and Pharmaceutical Research Unit, QU Health, Qatar University, P.O. Box 2713 Doha, Qatar

**Keywords:** resveratrol, COVID-19, inflammation, thrombosis, coagulation

## Abstract

Coagulation disorders, endotheliopathy and inflammation are the most common hallmarks in SARS-CoV-2 infection, largely determining COVID-19’s outcome and severity. Dysfunctions of endothelial cells and platelets are tightly linked in contributing to the systemic inflammatory response that appears to be both a cause and a consequence of COVID-19-associated coagulation disorders and thrombotic events. Indeed, elevated levels of circulating inflammatory cytokines are often associated with abnormal coagulation parameters in COVID-19 patients. Although treatments with low molecular weight heparin (LMWH) have shown beneficial effects in decreasing patient mortality with severe COVID-19, additional therapeutic strategies are urgently needed. Utilizing the anti-inflammatory and anti-thrombotic properties of natural compounds may provide alternative therapeutic approaches to prevent or reduce the risk factors associated with pre-existing conditions and comorbidities that can worsen COVID-19 patients’ outcomes. In this regard, resveratrol, a natural compound found in several plants and fruits such as grapes, blueberries and cranberries, may represent a promising coadjuvant for the prevention and treatment of COVID-19. By virtue of its anti-thrombotic and anti-inflammatory properties, resveratrol would be expected to lower COVID-19-associated mortality, which is well known to be increased by thrombosis and inflammation. This review analyzes and discusses resveratrol’s ability to modulate vascular hemostasis at different levels targeting both primary hemostasis (interfering with platelet activation and aggregation) and secondary hemostasis (modulating factors involved in coagulation cascade).

## 1. Introduction

On 11 March 2020, the Corona Virus Disease 2019 (COVID-19) was declared a global pandemic by the World Health Organization (WHO) (https://www.who.int/emergencies/diseases/novel-coronavirus-2019 (accessed on 06 February 2021)). The causative agent was determined to be the severe acute respiratory syndrome coronavirus 2 (SARS-CoV-2). This virus, which belongs to the Coronaviridae family, subfamily Orthocoronavirinae, has a positive single-stranded RNA genome and a characteristic crown-like spikes proteins on the outer surface [1]. Symptoms of COVID-19 are variable but the most common are fever, cough, breathing difficulties, as well as loss of smell and taste. Overall, most people affected by COVID-19 have mild to moderate symptoms and recover without special treatments, but older people and those with comorbidities, such as cardiovascular disease, diabetes, and chronic respiratory disease, are more likely to develop adverse outcomes [2].

The lungs are the most affected organs, probably because SARS-CoV-2 preferentially uses the angiotensin-converting enzyme 2 (ACE2) receptor, which is highly expressed in lung type II alveolar cells [3]. Indeed, a common condition of COVID-19 patients, often associated with high mortality rate, is the acute respiratory distress syndrome (ARDS), an acute and diffuse inflammatory lung injury marked by increased pulmonary vascular permeability and loss of aerated lung tissue [4]. In addition, the excessive intra-alveolar fibrin deposition, driven by an imbalance between activation of coagulation and inhibition of fibrinolysis, is a condition linked to ARDS pathophysiology [5]. Therefore, as a consequence of SARS-CoV-2 attack of cells in the pulmonary and vascular systems, COVID-19-patients with poor prognosis show the concurrence of ARDS with pulmonary vascular thrombosis and increased concentration of D-dimer, a small protein fragment resulting from clot degradation [6,7].

Overall, COVID-19 is a systemic and complex disease with a wide spectrum of clinical manifestations. Many COVID-19 patients show a severe proinflammatory state associated with both distinctive coagulopathy and procoagulant endothelial phenotype. Indeed, several lines of evidence suggest that the interconnection between coagulopathy and endotheliopathy can explain the microvascular and macrovascular thrombotic events that participate in the multiorgan dysfunctions in severe COVID-19 cases. [8,9]. Indeed, endotheliopathy, responsible for both the microvascular thrombotic events and the microcirculatory impairment observed in many COVID-19 patients, is a direct consequence of the virus endothelial infection and an indirect damage caused by the disease-associated inflammatory status [8,9]. Increased levels of Von Willebrand factor (vWF) and factor VIII (FVIII) as well as platelet hyperactivation characterize the endothelial status during COVID-19 [9].

COVID-19 is also a systemic inflammatory vascular disease, evident by the increased concentrations of proinflammatory cytokines in severe cases. These cytokines include tumor necrosis factor-α (TNF-α), interleukin 1 (IL1) and interleukin 6 (IL6), all of which are important regulators of coagulation [10]. In this context, treatment of COVID-19 patients with low molecular weight heparin (LMWH) has proven beneficial in reducing the risk of mortality resulting from thrombotic events [11,12]. However, since the use of these therapeutic anticoagulants is associated with increased bleeding, specific doses should be tuned to the patient’s overall condition [11,12,13,14]. Therefore, it remains crucial to develop additional anti-inflammatory, anticoagulant, or antithrombotic strategies to prevent and treat COVID-19.

There is an increasing need for alternative therapeutic approaches that can prevent or reduce the risk factors associated with both pre-existing conditions and comorbidities responsible for worsening COVID-19 patients’ outcomes [15]. Employing natural compounds characterized by anti-inflammatory and antithrombotic properties may provide an attractive avenue towards this goal. Among the several phytonutrients of interest, resveratrol (3,5,4′-trihydroxy-trans-stilbene), a polyphenol found in various plants, especially grapes, berries, peanuts, cacao and soybeans, may represent a good candidate [16]. This phytochemical possesses a wide range of biological activities, including anti-inflammatory, anticancer, antiviral, antioxidant, cardioprotective and neuroprotective [17]. Despite skepticism concerning its bioavailability and potential adverse effects, a crescent number of in vivo models showed the beneficial effects of resveratrol in several disease conditions [18,19]. Specifically, accumulating evidence supports the anti-inflammatory, anticoagulant and antithrombotic role of resveratrol [20,21,22]. Because of its polyvalent action in preventing or attenuating coagulation disorder, inflammation and vascular damage, major hallmarks of COVID-19, we believe resveratrol may be a good addition in the management of this disease. In this paper, we collect, review and critically discuss the evidence supporting the protective role that resveratrol might exert in ameliorating COVID-19-associated inflammatory conditions with particular emphasis on its antithrombotic actions.

## 2. Hemostatic Modulatory Properties of Resveratrol

**Figure 1:**
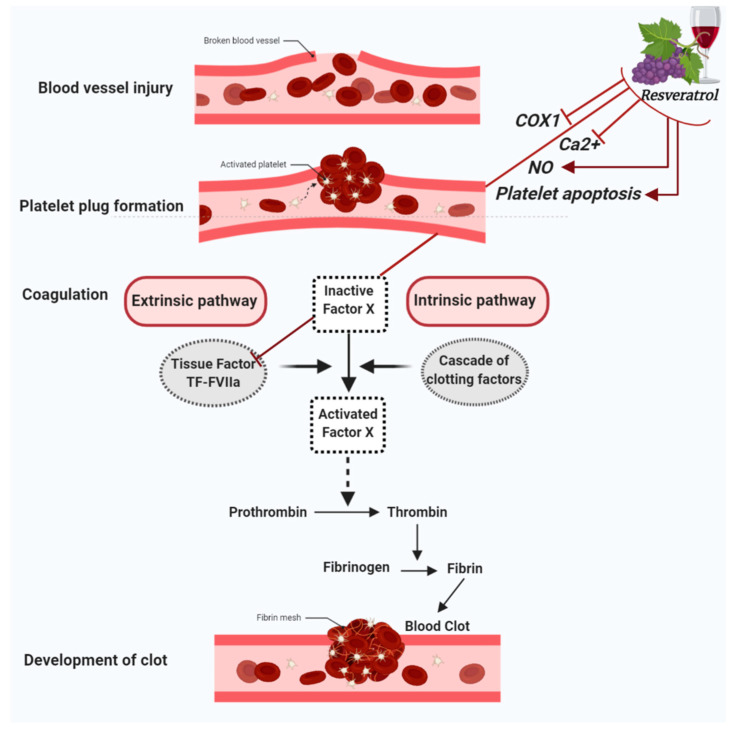
Figure summarizes the antiplatelet aggregation properties of resveratrol. Resveratrol inhibits cyclooxygenase-1 (COX-1), lowers nitric oxide (NO) concentration, decreases cytoplasmatic Ca^2+^ and blocks Ca^2+^ entry into platelets, which turn results in the suppression of platelet aggregation. Further antiplatelet aggregation properties of resveratrol are due to the activation of platelet apoptosis and the inhibition of Tissue factor (TF):FactorVIIa (FVIIa) complex (TF:FVIIa) formation.

When a blood vessel is injured, three mechanisms occur in a rapid sequence to control bleeding at the injury site: (1) a brief and intense vessel wall contraction, (2) formation of the platelet plug by platelet adhesion and aggregation, and (3) coagulation, which reinforces the platelet plug with fibrin. Platelet activation and aggregation in the initial plug is also called primary hemostasis. In contrast, secondary hemostasis refers to the coagulation cascade of enzymatic reactions that ultimately lead to the conversion of fibrinogen in fibrin monomers by the action of the clotting enzyme thrombin [23]. Furthermore, secondary hemostasis is traditionally divided into two pathways: the contact activation pathway (or intrinsic pathway) and the tissue factor pathway (or extrinsic pathway). In this regard, resveratrol has been shown to exert a protective role in vascular hemostasis by acting in both primary and secondary hemostasis. Indeed, besides having anti-platelet aggregation properties, resveratrol has also been shown to modulate factors involved in the coagulation cascade [20,21,22,24] (Figure 1).

### 2.1. Antiplatelet Aggregation Properties of Resveratrol

Platelet hyperactivity and aggregation contribute to thrombus formation and consequent blood vessels occlusion. In this regard, coagulopathy is observed in severe COVID-19 as a result of thrombus formation and consequent blood vessels occlusion [25,26]. Contextually, resveratrol showed anti-platelet aggregation properties that are exerted through different mechanisms. Like aspirin, resveratrol significantly inhibits cyclooxygenase-1 (COX-1), a key enzyme in the catalytic production of prostaglandins, which are key inflammatory mediators [27]. In platelets, the major product of COX-1 is thromboxane A_2_ (TXA_2_), a potent vasoconstrictor with prothrombotic properties capable of inducing platelet activation and aggregation [28]. Hence, inhibiting TXA_2_ production would promote blood flow and decrease clot formation. Similar to low doses of aspirin, resveratrol prevents thrombotic events via suppressing COX-1-derived TXA_2_ production in platelets [27,29].

Resveratrol can also suppress platelet aggregation by virtue of its ability to inhibit Ca^2+^ flux, which is known to largely determine the growth rate and extension of a thrombus [30,31]. In this context, resveratrol plays a dual-action by decreasing the release of Ca^2+^ from its stores as well as inhibiting Ca^2+^ entry into platelets and therefore their subsequent aggregation [32,33]. Another anti-platelet aggregation mechanism of resveratrol involves the nitric oxide (NO), a key gasotransmitter in physiological and pathological processes [34]. Indeed, NO is a potent vasodilator and an efficient thrombosis modulator that prevents platelet activation and aggregation [35]. Interestingly, it was recently proposed that modulating NO levels may be important in preventing, limiting or treating the severe pulmonary consequences of COVID-19 [36]. This is in accordance with reports showing that, besides its vasodilatorory and anti-inflammatory capacities, NO also has antiviral properties [37], and can indeed inhibit SARS-CoV-2 replication [38]. NO synthesis results from the action of three different nitric oxide synthase (NOS) isoforms, and the endothelial nitric oxide synthase (eNOS) is the isoform responsible for NO generation in the vascular endothelium [34]. In this regard, resveratrol appears to promote NO production via increasing eNOS expression and activity [39]. Specifically, some data show that resveratrol-elicited NO production result by its interacting with membrane-bound structures like the estrogen receptors [40,41]. Other works indicated that the resveratrol effect upon NO production is mediated by its direct interaction with intracellular pathways components including sirtuin-1 (SIRT1), adenosine monophosphate-activated protein kinase (AMPK) and nuclear factor erythroid 2-related factor 2 (Nrf2) [39]. NO bioavailability depends on the balance between ROS production and eNOS activity, since an increase in oxidative stress can alter eNOS function, whereby preferential shift towards production of superoxide rather than NO ensues [42]. The resulting imbalance between ROS and antioxidant defence mechanisms is the primary cause of endothelial dysfunction, a pathological endothelial condition characterized by a proinflammatory and procoagulant state [43]. This dysfunction, besides to emerging as a major player in SARS-CoV-2 infection [44,45], is the hallmark of comorbidities, such as hypertension, atherosclerosis, diabetes and obesity, which are often correlated with severe COVID-19 outcomes of [46,47]. In this context, since it upregulates NO synthesis while also decreasing ROS, resveratrol presents an attractive opportunity to be utilized. However, resveratrol can also reduce ROS levels through further mechanisms [48,49].

The functionality of endothelium is essential for maintaining hemostasis and preventing thrombosis. Indeed, an intact endothelium releases prostacyclin and nitric oxide, two vital vasoactive molecules that prevent platelet aggregation. On the other hand, insult-activated endothelial cells express a variety of molecules and receptors that increase platelet adhesion to the site of injury [50]. One of these molecules whose expression is turned on is tissue factor (TF), a protein known to be the primary cellular activator of blood coagulation. Indeed, after vessel injury or in response to inflammatory cytokines, TF forms the complex TF:FactorVIIa which in turn activates the extrinsic pathway of coagulation [51]. This may explain how TF might contribute or even drive COVID-19-associated coagulopathy. The vascular damage and cytokine storm that follow SARS-COV-2 infection of endothelial cells or monocytes might increase the expression of TF, which in turn aberrantly activates the coagulation cascade [52]. In endothelial and mononuclear cells, resveratrol strongly down-regulates TF expression by inhibition the c-Rel/p65/NF-κB pathway, a phenomenon which might partially explain the anticoagulant and antithrombotic properties of resveratrol [53].

Recently, it was shown that resveratrol can simultaneously inhibit platelet aggregation and stimulate platelet apoptosis [54]. Indeed, it can induce platelet apoptosis through both the extrinsic (cytoplasmic) and the intrinsic (mitochondrial) apoptotic pathways [54]. Due to this pro-apoptotic action in platelets, it would be only tempting to speculate that resveratrol may potentially work as an antithrombotic agent [54]. However, that remains to be investigated. (Figure 1).

### 2.2. Resveratrol Modulates Thrombotic Markers

Being heavily reported in histopathological analysis of large and small pulmonary vessels of COVID-19 patients, thrombosis is now widely recognized as a salient feature of the disease [55,56,57]. Venous thromboembolism (VTE) is another condition frequently observed in COVID-19 patients. Indeed, a recent systematic review from eighty-six studies reported that VTE occurs in both intensive care unit (ICU) and non-ICU hospitalized COVID-19 patients and it is often associated with increased d-dimer levels [58]. Several in vivo and in vitro studies highlighted resveratrol’s positive effect in reducing the incidence of venous thrombosis. In particular, resveratrol appears to modulate the expression of thrombosis-associated markers such as vWF, procoagulant factor VIII, and P-selectin [59,60,61]. Interestingly, levels of vWF and Factor VIII are directly linked to the severity of thrombosis and the ensuing stroke [62,63]. In addition, these appear to be common in COVID-19 patients, where they contribute to VTE [64,65]. Alarmingly, a young and asymptomatic SARS-CoV-2-infected patient who showed increased FVIII and vWF levels without any sign of hyperinflammatory state or blood coagulation activation suffered a stroke [66]. This provides additional evidence of the direct damage induced by SARS-CoV-2 to endothelial cells. Because of the COVID-19-induced damage, endothelium releases several molecules into the blood, including vWF, FVIII, tissue plasminogen activator-1 (t-PA-1) and P-selectin, which are usually stored within secretory organelles called Weibel–Palade bodies (WPB) that participate in platelet adhesion, secondary hemostasis and fibrinolysis [67]. P-selectin is required for the initial recruitment of leukocytes to the site of injury during inflammation and allows leukocytes interaction with activated endothelial cells and platelets [68]. On the other hand, t-PA-1 catalyzes the conversion of plasminogen to plasmin, the protein responsible for fibrin degradation in the process known as fibrinolysis, thus allowing the clot breakdown [69]. Since vWF, FVIII, t-PA-1 and P-selectin are directly linked to coagulopathy and endotheliopathy, two main features of COVID-19, one may consider them as biomarkers for the disease [8,65,70,71]. Importantly, resveratrol appears to decrease vWF, FVIII, t-PA-1 and P-selectin in HUVECs, suggesting a potential role in suppressing thrombus formation [59,60,61].

**Figure 2:**
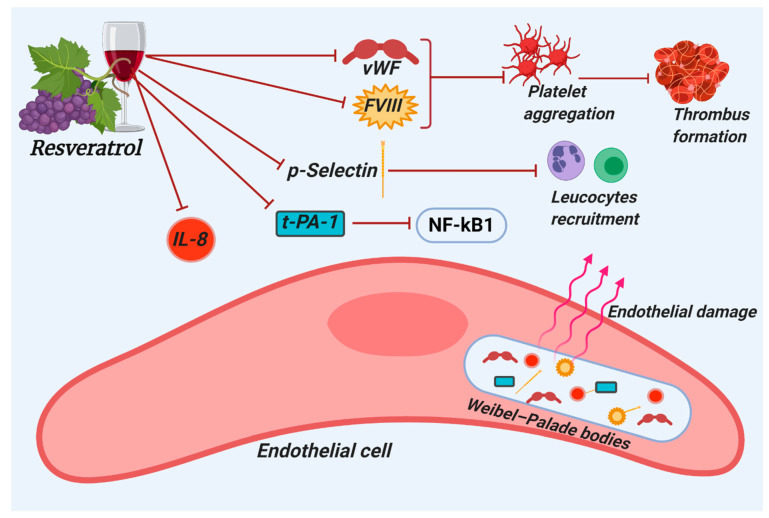
Figure summarizes the antithrombotic properties of resveratrol. Resveratrol counteracts the expression of thrombosis-associated markers such as Von Willebrand factor (vWF), factor VIII (FVIII), plasminogen activator-1 (t-PA-1), and P-selectin, which results in the inhibition of leucocytes recruitment, platelet aggregation and thrombus formation.

Resveratrol’s ability to mitigate inflammation has also been suggested, owing to its suppressive action on interleukin 8 (IL-8), a circulating inflammatory cytokine. The relationship between increased proinflammatory cytokines and coagulopathy is intricate and has emerged as key player in the pathogenesis of COVID-19 [72]. Moreover, not only host factors have an impact on COVID-19-associated hemostatic disorders, but also specific viral proteins can significantly trigger the increase in pro-inflammatory cytokines, and consequently activation of the coagulation system [73]. For instance, the viral nucleocapsid protein (N) can activate cyclooxygenase-2 (COX-2) [74], whereas the envelope protein (E) affects the production of pro-inflammatory cytokines such as TNF, IL-1 and IL-6 [75]. IL-6, along with IL-1β and IL-8, precipitates a systemic inflammatory milieu that then evokes both hypercoagulation and platelet hyper-activation [76,77]. Resveratrol appears to sufficiently suppress expression of these interleukins [78,79,80] as well as that of NF-κB [81]. Taken together, these findings further support the potentiality of resveratrol as a coadjutant in the treatment of inflammatory conditions such as the COVID-19-associate cytokine storm [82]. Indeed, in vascular inflammation, the activation of NF-κB signaling leads to the production of IL-6 [83]. Interestingly, it was recently shown that SARS-CoV-2-potentiated NF-κB activation in the lung alveoli elicits a state of uncontrolled inflammation leading to downstream organ failure [84]. Finally, as mentioned above, resveratrol decreases the secretion of t-PA-1 that, besides acting in fibrinolysis, is also an activator of NF-κB [61]. Resveratrol therefore, acting on t-PA-1, exerts an indirect anti-inflammatory action toward NF-kB. The protective effects of resveratrol have been confirmed by in vivo studies showing a reduction in PVT in rats, probably due to the resveratrol’s anti-platelet aggregation properties [85,86] (Figure 2).

## 3. Conclusions

By virtue of its ability to modulate platelet activation and aggregation as well as factors involved in the coagulation cascade, resveratrol appears to be an attractive pharmacotherapeutic agent in the fight against COVID-19. We speculate that it could serve as an adjunct treatment for slowing and ameliorating phenomena associated with the severe COVID-19 outcomes, such as vascular thrombosis and systemic inflammation. A limitation in the use of resveratrol is its poor bioavailability and rapid metabolism that might require an increased oral dose administration [19]. In this regard, human clinical trials have found resveratrol generally well-tolerated at doses up to 5 g/day although the occurrence of mild to moderate side effects suggests the use of a significantly lower dose [19,87,88]. Resveratrol doses between 100 and 200 mg/day showed beneficial effects on stroke major risk factors such as blood pressure, weight status, glucose, and lipid profile [89]. Taken at weekly intervals by overweight/obese individuals with mildly elevated blood pressure, resveratrol at doses of 30, 90, and 270 mg/day, elicited an improvement of the flow-mediated dilatation of the brachial artery (FMD), a widely recognized biomarker of endothelial function and cardiovascular health [90]. Although a dosage ranging between 30 and 300 mg would appear to maximize resveratrol benefits while minimizing potential side effects, further clinical trials are needed to provide definitive answers in this context.
